# Genetic and immune identification and functional analysis of TRPM8 as a potential biomarker for pancreatic adenocarcinoma proliferation

**DOI:** 10.1002/cnr2.2108

**Published:** 2024-06-04

**Authors:** Sen Qiao, Fengming Wu, Hongmei Wang

**Affiliations:** ^1^ Assisted Reproduction Center Northwest Women's and Children's Hospital Xi'an China; ^2^ School of Medicine Southeast University Nanjing Jiangsu China; ^3^ Shaanxi University of Chinese Medicine Xianyang China

**Keywords:** bioinformatic, pancreatic adenocarcinoma, prognostic markers, transient receptor potential channel, TRPM8

## Abstract

**Background:**

Pancreatic adenocarcinoma (PAAD), a member of highly lethal malignant tumors, has a poor outcome and extremely poor prognosis. The transient receptor potential (TRP) superfamily, a group of nonselective cation channels, is capable of influencing cellular functions by regulating calcium homeostasis. In addition, it has been shown that TRP channels can also affect various cellular phenotypes by regulating gene transcription levels and are involved in the development of a variety of malignant tumors.

**Aims:**

In order to find new therapeutic targets and biomarkers to improve the clinical prognosis of pancreatic cancer, we performed genetic and immunological characterization of TRP channels in PAAD, as well as related functional and prognostic analyses.

**Methods and Results:**

We investigated the expression, genetic alterations, methylation levels, and immune infiltration levels of TRP channels in PAAD, and further also analyzed the function of TRP channels in PAAD and their prognostic value for PAAD patients. Our results suggest that TRPM8 may contribute to tumor proliferation by controlling the PI3K‐AKT–mTOR signaling pathway in PAAD.

**Conclusion:**

After careful evaluation of the accumulated data, we concluded that TRPM8 has potential as a prognostic indicator and prospective therapeutic target in PAAD.

## 1 INTRODUCTION

The transient receptor potential (TRP) channel superfamily is a collection of nonselective cation channels, encompassing seven subfamilies: TRPC, TRPV, TRPM, TRPN, TRPA, TRPP, and TRPML.^1^ TRP channels play a significant role in maintaining calcium homeostasis and regulating related cellular processes by modulating Ca^2+^ ion flow.^2^ It has been observed that TRP channels possess the capacity to influence the cell cycle by regulating gene transcription levels, thereby impacting a range of cellular phenotypes such as cell proliferation, apoptosis, and cell motility.^3^ A growing body of research has elucidated the substantial contribution of the TRP channel superfamily to the progression and development of various cancer types.

Notably, the reduction in TRPC1 expression in nasopharyngeal carcinoma cells has been found to markedly attenuate their migratory and invasive capabilities.^4^ Furthermore, the application of siRNA to suppress TRPC1 expression in lung cancer cells has been shown to induce cell cycle arrest, consequently, impeding cell proliferation.^5^ The overexpression of TRPV6 has been confirmed in the malignant progression of diverse tissues, including thyroid cancer, ovarian cancer, K‐562 chronic myeloid leukemia cells, and the SW480 colorectal cancer cell line.^6^, ^7^ Moreover, TRPV1 also contributes to the progression of cancer. Particularly, the expression of TRPV1 is significantly increased in colon, bladder, and prostate cancers. The proliferation of MCF‐7 breast cancer cell lines is hindered by both agonists and antagonists of TRPV1,^8^ although the underlying mechanism is not well understood. Furthermore, TRPM7 is implicated in gastric cancer, pancreatic cancer, prostate cancer, ovarian cancer, and human hypopharyngeal squamous cell carcinoma.^9^ Moreover, in addition to its carcinogenic properties, specific TRP channels have been observed to promote the apoptosis of cancer cells. Studies have revealed an inverse correlation between the expression levels of TRPM1 and the metastasis of melanoma,^10^ and the absence of TRPM1 in cancer cells results in uncontrolled cell proliferation. Thus, it is apparent that the TRP family, operating as a comprehensive superfamily of ion channels, plays a substantial role in the advancement of various malignant cancers. Therefore, it is imperative to conduct a comprehensive and thorough investigation into their additional roles in cancer.

Pancreatic cancer, specifically ductal adenocarcinoma, is recognized as one of the most lethal malignancies, ranking within the top five. It is associated with an exceedingly poor prognosis.^11^ In recent times, there has been a consistent increase in the occurrence and mortality rates of this disease worldwide. Despite its relatively low incidence, pancreatic cancer demonstrates an alarmingly high fatality rate. Chemotherapy and radiotherapy are considered the primary therapeutic interventions, while surgical resection remains the most efficacious curative strategy.^12^ Nevertheless, the efficacy of surgical resection for patients with advanced pancreatic cancer remains significantly inadequate, resulting in a near‐impossibility of achieving a cure. Additionally, the absence of a universally accepted and reliable screening test for pancreatic cancer necessitates the clinical application of biomarkers in conjunction with imaging analysis techniques. Notably, carbohydrate antigen (CA)19‐9 and carcinoembryonic antigen (CEA) in the pancreatic fluid are frequently employed as biomarkers in clinical practice.^13^, ^14^ However, it is crucial to acknowledge that CA19‐9 demonstrates increased levels exclusively in 65% of pancreatic cancers and biliary tract disease.^15^ Oncogenes, tumor suppressors, and adipokines were addressed and studied extensively as markers for pancreatic cancer, such as lipocalcins, with or without diabetes as a risk complicating factor, IGF (insulin‐like growth factor), MIF (migration inhibitory factor), and ZIP4 (a zinc ion transporter).^16^, ^17^, ^18^ However, the role of the TRP family in pancreatic cancer remains to be investigated. Heightened levels of the TRP family have been detected in malignancies originating from diverse organs, encompassing biliary obstruction. Nevertheless, as a biomarker for pancreatic cancer, the sensitivity and specificity of TRP family markers are relatively diminished. Likewise, the CEA also presents limitations, displaying high specificity but low sensitivity. Currently, the potential of the TRP family as viable biomarkers or therapeutic targets for pancreatic cancer remains uncertain. Relevant studies have shown that this could be achieved via epigenetics markers long noncoding RNAs and microRNAs.^19^, ^20^ In view of the above background, TRP channels play a role in a variety of malignant tumors, and the challenges and urgency posed by the poor efficacy and very poor prognosis of pancreatic adenocarcinoma (PAAD). In order to further fill the lack of studies related to TRP channels in pancreatic cancer, and to find new therapeutic targets and biomarkers to improve the clinical prognosis of pancreatic cancer, we performed genetic and immunological characterization of TRP channels in PAAD, as well as related functional and prognostic analyses. In order to clarify the potential of the TRP family as a viable biomarker or therapeutic target for pancreatic cancer and to assist in the clinical diagnosis and treatment of pancreatic cancer patients, this study was conducted to contribute to TRP therapeutic efficacy in the context of pancreatic cancer, consequently.

## 2 MATERIALS AND METHODS

### 2.1 UALCAN (https://ualcan.path.uab.edu/analysis.html)

UALCAN is a comprehensive web‐based tool that facilitates the retrieval and examination of diverse cancer histology data sets, including The Cancer Genome Atlas (TCGA), MET500, and CPTAC.^21^ Utilizing the gene expression analysis functionalities provided by UALCAN, we have conducted an investigation into the contrasting expression patterns of the TRP family in PAAD and healthy tissues.

### 2.2 GEPIA (http://gepia.cancer-pku.cn/)

The GEPIA database, which comprises sequencing data from 9736 cancer tissues and 8587 normal paracancer tissues sourced from the medium GTEx database and the TGAC database, provides users with the capability to conduct expression analysis, survival analysis, and downscaling analysis.^22^ In this study, we utilized the GEPIA database to investigate the potential association between the TRP family and the pathological stage of patients with PAAD. Additionally, we examined the expression patterns of the TRP family in PAAD.

### 2.3 Kaplan–Meier plotter (https://kmplot.com/analysis/)

The Kaplan–Meier plotter database facilitated the evaluation of the correlation between gene expression data and prognostic significance across a comprehensive set of 21 different cancer types. Through the utilization of this database, researchers conducted survival analyses specifically targeting genes of interest. In this study, we employed the database to conduct an overall survival (OS) analysis and recurrence‐free survival analysis for the TRP family in PAAD.

### 2.4 MethSurv (https://biit.cs.ut.ee/methsurv/)

The MethSurv database encompasses a comprehensive collection of 7538 methylation data points derived from 25 distinct cancer types. This database offers the capability to conduct both univariate and multivariate survival analyses utilizing DNA methylation data.^23^ In the present study, we employed this database to investigate the methylation levels of the TRP family.

### 2.5 SurvivalMeth (https://bio.tools/survivalmeth#!)

SurvivalMeth is a novel database that facilitates the investigation of the prognostic implications of DNA methylation. It encompasses diverse datasets from TCGA, GEO, and CCLE, encompassing a wide range of DNA methylation features, such as 309 465 CpG island‐associated elements, 104 748 transcription‐associated elements, 77 634 repeat elements, and more.^24^ In this study, we employed this comprehensive database to assess the influence of TRP family methylation on the prognosis of PPAD patients.

### 2.6 cBioPortal (https://www.cbioportal.org/)

The cBioPortal website serves as a platform for the integration of data derived from 126 tumor genomic studies conducted within the TCGA and ICGC large‐scale oncology research projects, encompassing a total of 28 000 distinct specimens. This resource facilitates the visual analysis of multidimensional cancer genomics data, specifically enabling the examination of mutations within the TRP family among patients diagnosed with PAAD.^25^

### 2.7 String (https://cn.string-db.org/)

String, an online database renowned for its capacity to analyze protein–protein interactions (PPIs), provides PPI networks alongside robust visualization capabilities. In our study, we employed this database to investigate the interactions occurring within the TRP families.

### 2.8 GeneMANIA (http://genemania.org/)

GeneMANIA is a database generating hypotheses about gene function, analyzing gene lists, and analyzing gene priorities based on function.^26^ We used it to analyze neighboring genes that interact with the TRP family.

### 2.9 Bioinformatics (http://www.bioinformatics.com.cn/)

Bioinformatics is a powerful online bioinformatics analysis website that enables rapid processing and visualization of many types of research data. We used this site to visualize and analyze the GO and KEGG pathways of the TRP family.

### 2.10 Timer (http://timer.cistrome.org/)

The Timer online analysis website uses RNA‐Seq expression profiling data to detect immune cell infiltration in different tumor tissues and also analyze the impact of immune infiltration on clinical prognosis.^27^ We used this database to analyze the correlation between the TRP family and immune cell infiltration and the impact on prognosis.

### 2.11 GDSC database (https://www.cancerrxgene.org/)

We have utilized the Cancer Drug Sensitivity Genomics (GDSC), the most comprehensive publicly accessible pharmacogenomics database in the field of oncology. This resource, available at https://www.cancerrxgene.org/, employs transcriptomic data derived from individual samples to anticipate the response to drug treatment for each respective sample.

### 2.12 Gene expression assays

For quantitative Reverse Transcription‐Polymerase Chain Reaction (RT‐PCR)c, samples including para‐carcinoma tissues (PARA) and PAAD tissues (aged between 30 and 60; no gender limitation) were dissolved in Trizol (Invitrogen, Carlsbad, CA), and RNA isolation was performed in accordance with the manufacturer's instructions. The BluePrint RT reagent kit (Takara, Tokyo, Japan) was utilized for reverse transcription of 1 μg RNA. The CFX96 Real‐time System (Bio‐Rad) and SYBR Green Supermix (Bio‐Rad) were employed for cDNA level quantification via quantitative PCR. Variations in gene expression were ascertained by comparing cDNA quantities in the target (treated) samples to those in a calibrator sample (vehicle). The details of primers were shown in Table S1.

### 2.13 Statistics

Quantitative data are expressed as mean ± standard deviation (SD). For comparison between two groups, Student's *t*‐test was implemented. When comparing more than two groups, one‐way ANOVA with Tukey's post hoc test was employed. Statistical significance was acknowledged at a *p*‐value of less than .05.

## 3 RESULTS

### 3.1 Analysis of the TRP family in PAAD

**FIGURE 1:**
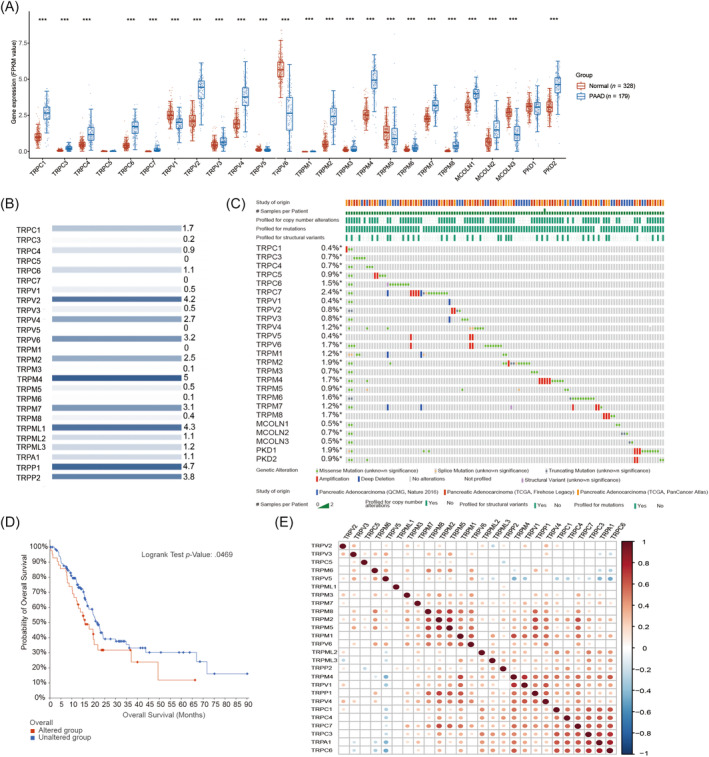
The relative expression levels and genetic alterations of all transient receptor potentials (TRPs) in pancreatic adenocarcinoma (PAAD). (A) The expression patterns of TRP family members in normal and PAAD groups (the cancer adjacent samples in the TCGA database and their corresponding pancreatic cancer tumor tissues). The asterisk denotes the significance level (*p), where *p < .05, **p < .01, and ***p < .001. The significance comparison between two sample groups is assessed through the Wilcoxon test, whereas the significance comparison among three sample groups is conducted using the Kruskal–Wallis test. (B) Genetic alterations in TRPs with cBioPortal. The blue hue signifies the proportional expression magnitude of TRP channels in PAAD, wherein a darker shade of blue corresponds to elevated expression levels of TRP channels in PAAD. (C) The relative expression level of TRPs in PAAD (GEPIA). (D) The impact of all TRP family mutations on the prognosis of PAAD patients. (E) The correlation between TRP family members using SurvialMeth.

Initially, the expression of the TRP family in PAADand normal tissues was examined using the UALCAN database. It was observed that TRPC1, TRPC4, TRPC6, TRPV2, TRPV4, TRPM2, TRPM8, and PKD2 exhibited elevated expression levels in PAAD. Conversely, the expression of TRPV1 and TRPV6 was found to be low in PAAD patients (Figure 1A). Subsequently, the relative expression levels of the TRP family in PAAD were analyzed using the GEPIA database, revealing that TRPM4 displayed the highest expression level in PAAD. Simultaneously, TRPC5, TRPC7, TRPV5, and TRPM1 were observed to be minimally expressed in PAAD (Figure 1B). The genetic alterations of the TRP family in 179 patients with PAAD were examined using the cBioPortal database. The analysis revealed that TRPC (1, 3–7), TRPV (1–6), TRPM (1–8), MCOLN (1–3), and PKD (1, 2) exhibited genetic alterations in PAAD patients, with frequencies ranging from 0.4% to 2.4%. These alterations were primarily observed in missense mutations and amplifications. Notably, TRPC6, TRPC7, TRPV6, TRPM2, TRPM4, TRPM6, TRPM8, and PKD1 displayed a particularly high mutation rate, exceeding 1.5% (Figure 1C). The subsequent analysis involved an examination of the influence of TRP family mutations on the prognosis of patients with PAAD. It was observed that patients harboring alterations (the genetically altered TRPs are detailed in Figure 1C) in the TRP family exhibited a poorer prognosis, although the statistical significance was not established (Figure 1D). In the initial stage, the correlation between TRP family members in PAAD was investigated utilizing SurvialMeth (Figure 1E). Notably, a robust correlation was observed between TRPCs and TRPMs, especially, TRPM8 and TRPV6. Further elaboration on this matter can be found in Figures 1E and S1A.

The analysis of correlation and PPI networks among TRP families was conducted. Initially, the correlation between members of the TRP family was examined using the SurvialMeth tool (Figure S1B). It was observed that TRPV1 exhibited a significant correlation with PDK1, with a coefficient of .039 (*p*‐value). Additionally, a strong correlation was observed between TRPM8 and TRPM5. Subsequently, the interactions between TRP family members were investigated using the String database, revealing that TRPV1 is associated with a majority of other TRP family members, such as TRPV2, TRPA1, TRPM8, TRPV3, and others (Figure S1C). ITPR3 encodes the receptor for inositol 1,4,5‐trisphosphate, which serves as a mediator for intracellular Ca^2+^ release as a second messenger. Additionally, the TRP family was subjected to GO‐KEGG enrichment analysis. The results of the GO analysis revealed that the TRP family primarily participates in various biological processes, including Ca^2+^ transport across the membrane, Ca^2+^ release, regulation of Ca^2+^ concentration in the cell membrane, protein tetramerization, and others. Furthermore, the TRP family is also involved in the formation of the cation channel complex, polycystin complex, and the composition of the pathway, as depicted in Figure 1G. KEGG enrichment results showed that the TRP family is closely related to regulating inflammatory mediators and mineral uptake (Figure S1D).

### 3.2 Analysis of the prognostic value of the TRP family in PAAD patients

**FIGURE 2:**
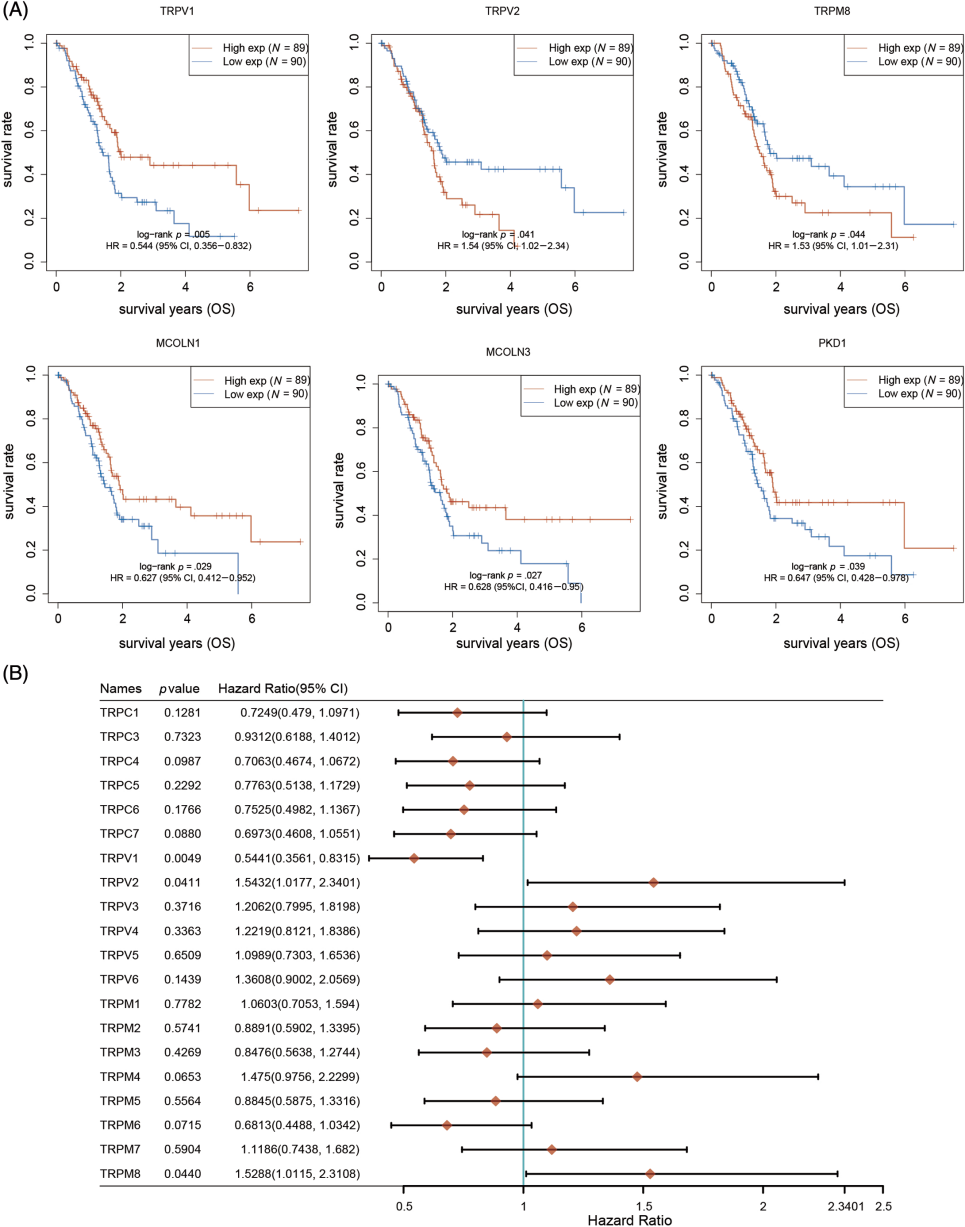
Overall survival (OS) for the expression of transient receptor potentials (TRPs) in pancreatic adenocarcinoma (PAAD) patients. (A) The OS curve of TRPV1, TRPV2, TRPM8, MCOLN1, MCOLN2, and PKD1. (B) The hazard ratio (HR) of TRP family members about prognosis in PAAD patients. Raw counts of RNA‐sequencing data (level 3) and corresponding clinical information from PAAD were retrieved from The Cancer Genome Atlas (TCGA) dataset (https://portal.gdc.cancer.gov/), following guidelines and policies for data acquisition and utilization. The Kaplan–Meier (KM) survival analysis with log‐rank test was employed to assess the survival disparity between the aforementioned two groups. For the KM curves, *p*‐values and HRs with 95% confidence intervals (CI) were calculated using log‐rank tests and univariate Cox proportional hazards regression. All statistical analyses and R packages were executed using R software version v4.0.3 (The R Foundation for Statistical Computing, 2020). A significance threshold of *p* < .05 was applied for determining statistical significance.

**FIGURE 3:**
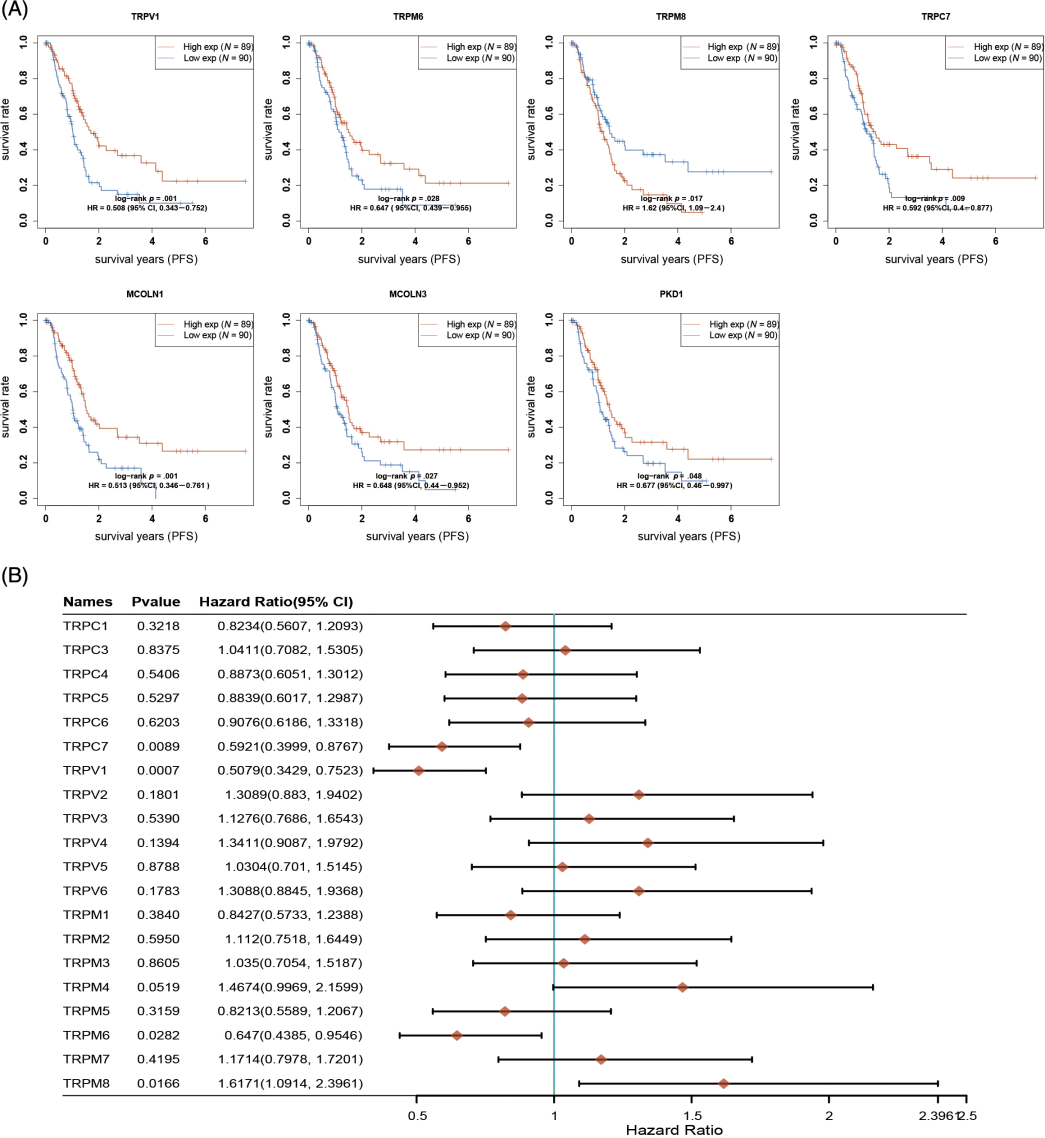
Progression‐free survival (PFS) for the expression of transient receptor potential (TRPs) in pancreatic adenocarcinoma (PAAD) patients. (A) The overall survival curve of TRPV1, TRPM6, TRPM8, TRPC7, MCOLN1, MCOLN2, and PKD1. (B) The hazard ratio of TRP family members about prognosis in PAAD patients.

**FIGURE 4:**
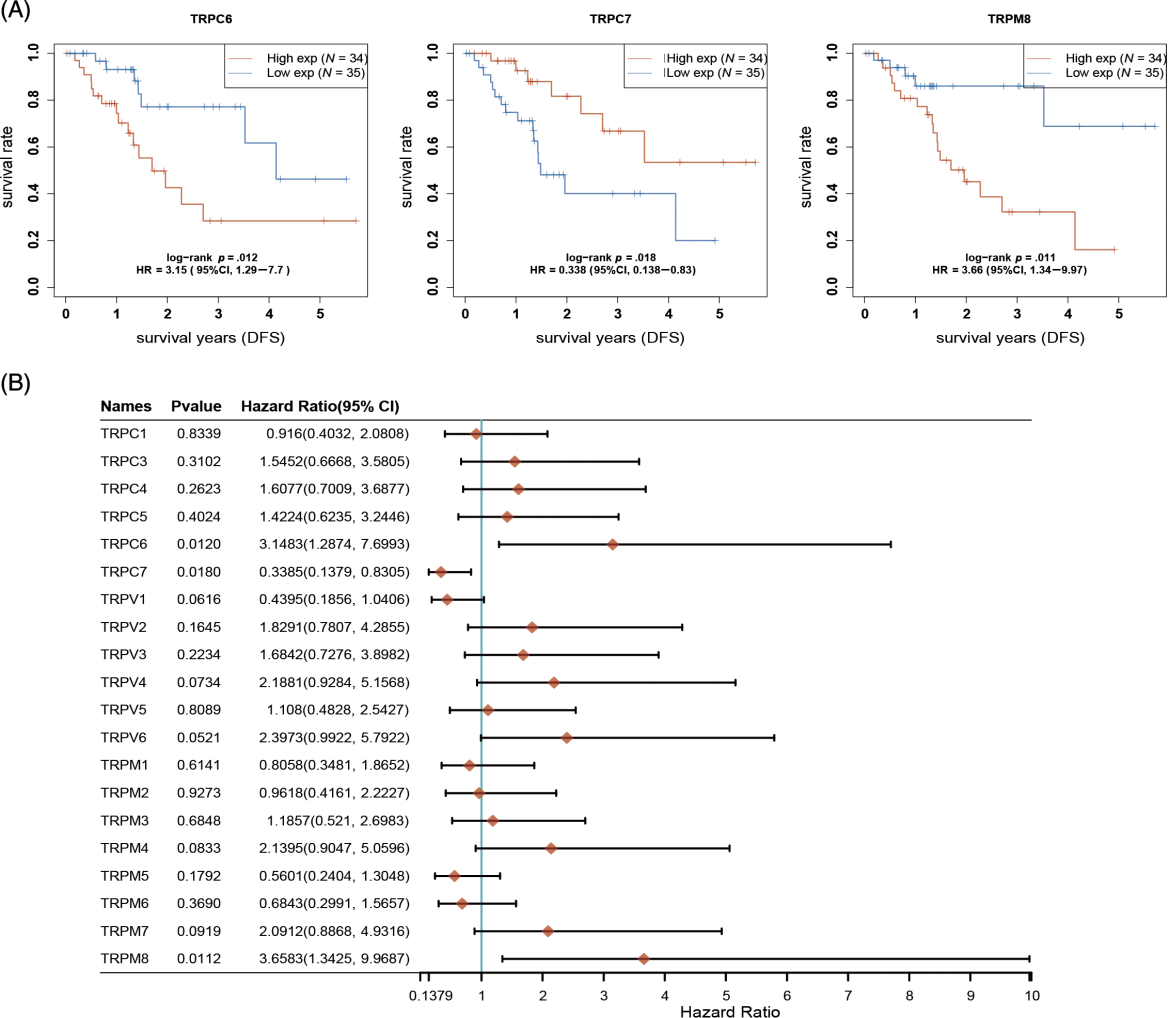
Disease‐free survival (DFS) for the expression of transient receptor potentials (TRPs) in pancreatic adenocarcinoma (PAAD) patients (Kaplan–Meier plotter). (A) The overall survival curve of TRPC6, TRPC7, and TRPM8. (B) The hazard ratio (HR) of TRP family members about prognosis in PAAD patients.

**FIGURE 5:**
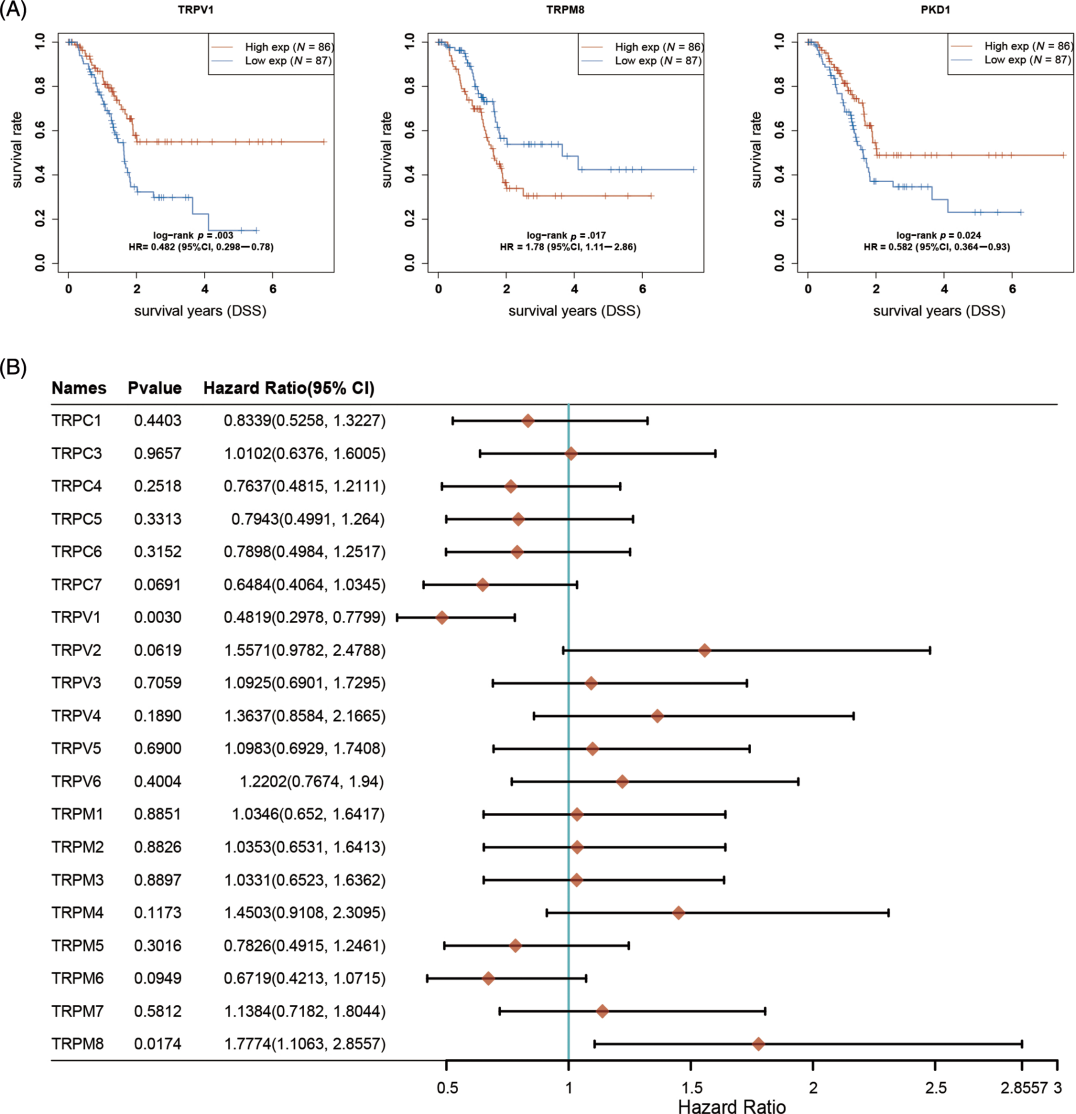
Disease‐free survival (DSS) for the expression of transient receptor potentials (TRPs) in pancreatic adenocarcinoma (PAAD) patients (Kaplan–Meier plotter). (A) The overall survival curve of TRPV1, TRPM8, and PKD1. (B) The hazard ratio (HR) of TRP family members about prognosis in PAAD patients.

The prognostic impact of the TRP family in patients with PAAD was investigated using the Kaplan–Meier plotter online analysis website. The analysis revealed that patients with low expression of TRPV1, TRPML1 (MCOLN1), TRPML3 (MCOLN3), and TRPP1 (PKD1) had significantly shorter OS, while those with high expression of TRPV2 and TRPM8 had a poorer prognosis (Figure 2A). Furthermore, the hazard ratio (HR) of patients with PAAD was analyzed based on their OS. The results indicated that TRPV (2–6), TRPM1, TRPM4, TRPM7, and TRPM8 were identified as risk factors influencing the development of PAAD (Figure 2B). According to the progression‐free survival (PFS) curves, patients exhibiting low levels of TRPV1, TRPM6, TRPC7, TRPML1, TRPML3, and TRPP1 experienced a more unfavorable prognosis following antitumor therapy. Conversely, patients with high expression of TRPM8 demonstrated a poorer prognosis after antitumor therapy compared with those with low expression of these genes (Figure 3A). By examining the PFS of patients, we conducted an analysis of the HR among individuals with PAAD. The data depicted in Figure 3B indicate that TRPC3, TRPV (2–6), and TRPM (2–4, 7, 8) serve as risk factors influencing the development of PAAD. In order to assess the impact of TRPs channel on the prognosis of PAAD, we conducted an analysis on the correlation between the alteration in TRPs channel expression and the disease‐free survival (DFS) of PAAD patients. DFS denotes the duration from randomization to either disease recurrence or mortality from any cause. Our findings indicate that elevated levels of TRPC6 and TRPM8 expression are associated with a deteriorated DFS among patients (Figure 4A). Conversely, patients with diminished TRPC7 expression exhibited a poorer prognosis. We conducted further analysis on the HR using DFS and identified TRPC (3–6), TRPV (2–6), and TRPM (3, 4, 7, 8) as significant risk factors for DFS in patients with PAAD (Figure 4B). Additionally, we investigated the impact of changes in TRPs channel expression on disease‐specific survival (DSS) in PAAD patients, which refers to the time of death caused by the specific disease. We observed that patients with low expression of TRPV1 and PKD1 had a poorer prognosis, while higher expression of TRPM8 was associated with worse DSS in patients (Figure 5A). The HR analysis conducted in this study revealed that TRPC3, TRPV (2–6), and TRPM (1–4, 7, 8) were identified as risk factors for DSS in patients with PAAD (Figure 5B). Furthermore, the HR analysis of patients' OS, PFS, DFS, and DSS indicated that TRPM8 was a statistically significant risk factor impacting patients' OS, PFS, DFS, and DSS (*p* < .05). In summary, higher expression levels of TRPM8 were associated with a poorer prognosis in patients. Moreover, our examination utilizing the International Cancer Genome Consortium (ICGC) database demonstrated a correlation between elevated TRPM8 expression and poorer OS rates in patients diagnosed with PAAD (Figure S2). This evidence aligns with the outcomes derived from our preceding analysis of TCGA database.

### 3.3 Methylation levels of TRP family in PAAD and prognostic analysis

**FIGURE 6:**
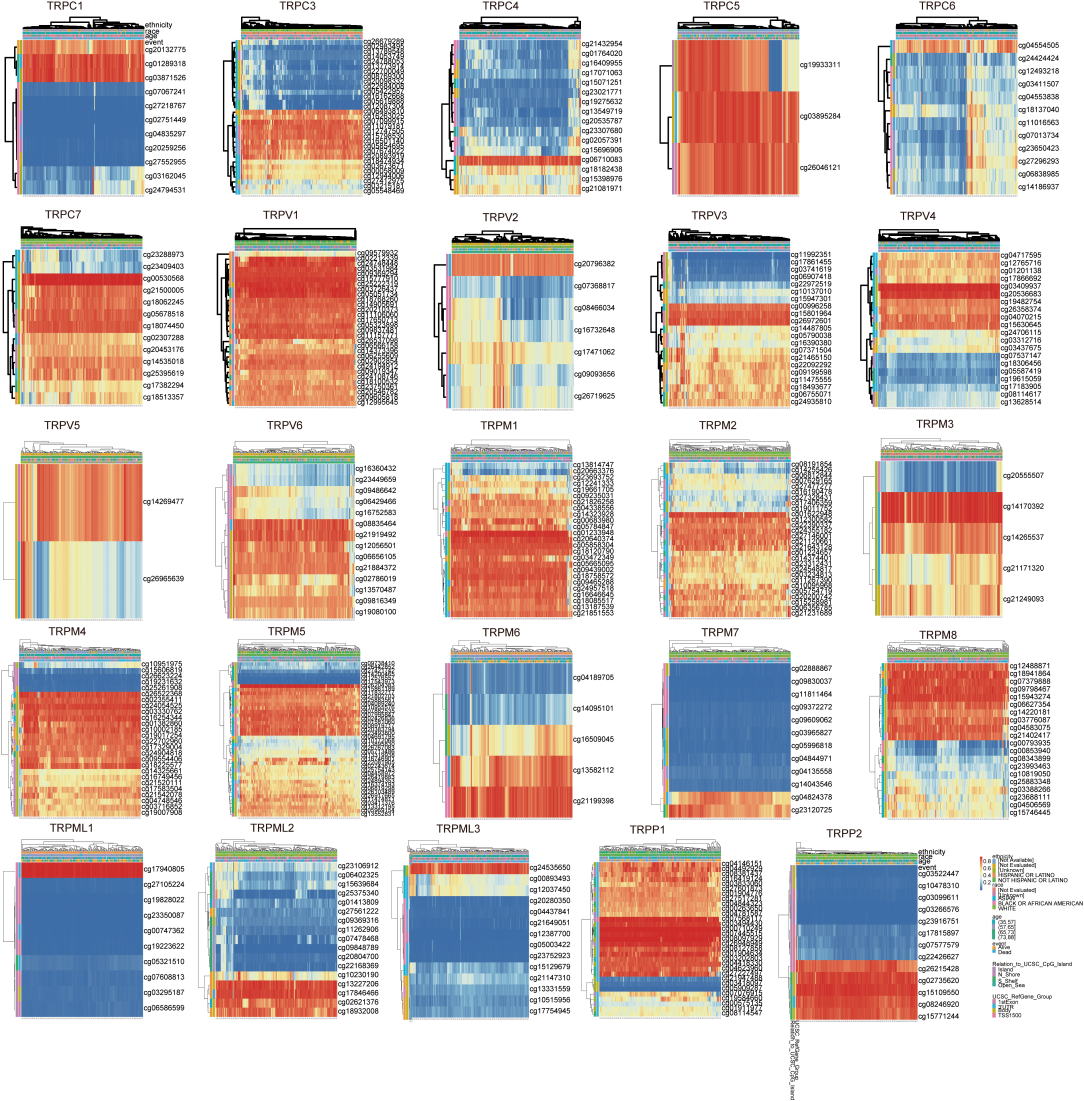
Methylation levels of transient receptor potentials (TRPs) family in pancreatic adenocarcinoma (PAAD) and prognostic analysis. The methylation levels of the TRP family in PAAD was used the MethSurv database.

We analyzed the methylation levels of the TRP family in PAAD using the MethSurv database (Figure 6). We found cg03871526 for TRPC1, cg070999115 for TRPC3, cg06710083 for TRPC4, cg03895284 for TRPC5, andTRPC6's cg04554505, TRPC7's cg00530568, TRPV1's cg15777910, TRPV2's cg20796382. TRPV3's cg15801964, TRPV4's cg03409937, TRPV5's cg14269477, TRPV6's cg21919492.TRPM1's cg01233948, TRPM2's cg01622948, TRPM3's cg14170392, TRPM4's cg24054525, TRPM5's cg26204383, TRPM6's cg21199398, TRPM7's cg23120725, TRPM8's cg15943274, cg17940805 for TRPML1, cg17846466 for TRPML2, cg24535650 for TRPML3, cg25229089 for TRPA1, cg00710249 for TRPP1, and cg15109550 for TRPP2 had the highest level of site methylation. The comprehensive details are enumerated in Table 1, wherein the statistical significance of cg15943274 in TRPM8 is notably substantial.

**TABLE 1:** Methylation sites of the TRP family in PAAD.

Gene	Relation to island	Genomic region	Methylation sites	Best split	Correlation coefficient^a^	*p* value
TRPC1	Island	Body	cg03871526	Median	−0.048	0.0012
TRPC3	Island	Body	cg070999115		−0.241	0.013
TRPC4	Open_Sea	Body	cg06710083		0.069	0.0035
TRPC5	N_Shore	5'UTR	cg03895284		−0.051	0.023
TRPC6	Island	TSS200	cg04554505		0.021	0.0026
TRPC7	Island	Body	cg00530568		0.195	0.0035
TRPV1	Island	Body	cg15777910		0.215	0.0024
TRPV2	Open_Sea	Body	cg20796382		0.208	0.00019
TRPV3	Open_Sea	TSS200	cg15801964		0.307	0.00009
TRPV4	Open_Sea	Body	cg03409937		−0.042	0.018
TRPV5	Open_Sea	5'UTR;1stExon	cg14269477		−0.152	0.049
TRPV6	Open_Sea	Body	cg21919492		0.107	0.011
TRPM1	Island	Body	cg01233948		0.101	0.013
TRPM2	N_Shelf	Body	cg01622948		−0.062	0.0029
TRPM3	Open_Sea	Body	cg14170392		0.102	0.015
TRPM4	S_Shore	Body	cg24054525		0.217	0.0046
TRPM5	Island	Body	cg26204383		−0.005	0.0081
TRPM6	Open_Sea	3'UTR	cg21199398		0.203	0.0026
TRPM7	Island	1stExon;5'UTR	cg23120725		−0.047	0.017
TRPM8	Open_Sea	Body	cg15943274		0.102	0.0047
TRPML1	Island	TSS200	cg17940805		0.017	0.0026
TRPML2	Island	TSS1500	cg17846466		−0.005	0.0011
TRPML3	N_Shelf	Body	cg24535650		0.203	0.0015
TRPA1	Island	1stExon;5'UTR	cg25229089		−0.047	0.0021
TRPP1	N_Shore	Body	cg00710249		0.138	0.0015
TRPP2	N_Shore	TSS1500	cg15109550		0.176	0.0058

Abbreviations: PAAD, pancreatic adenocarcinoma; TRP, transient receptor potential.

^a^ Adjustments made following variables such as age, gender, race, and pathological stage.

### 3.4 Correlation analysis between TRP family and immune cell infiltration in PAAD patients

**FIGURE 7:**
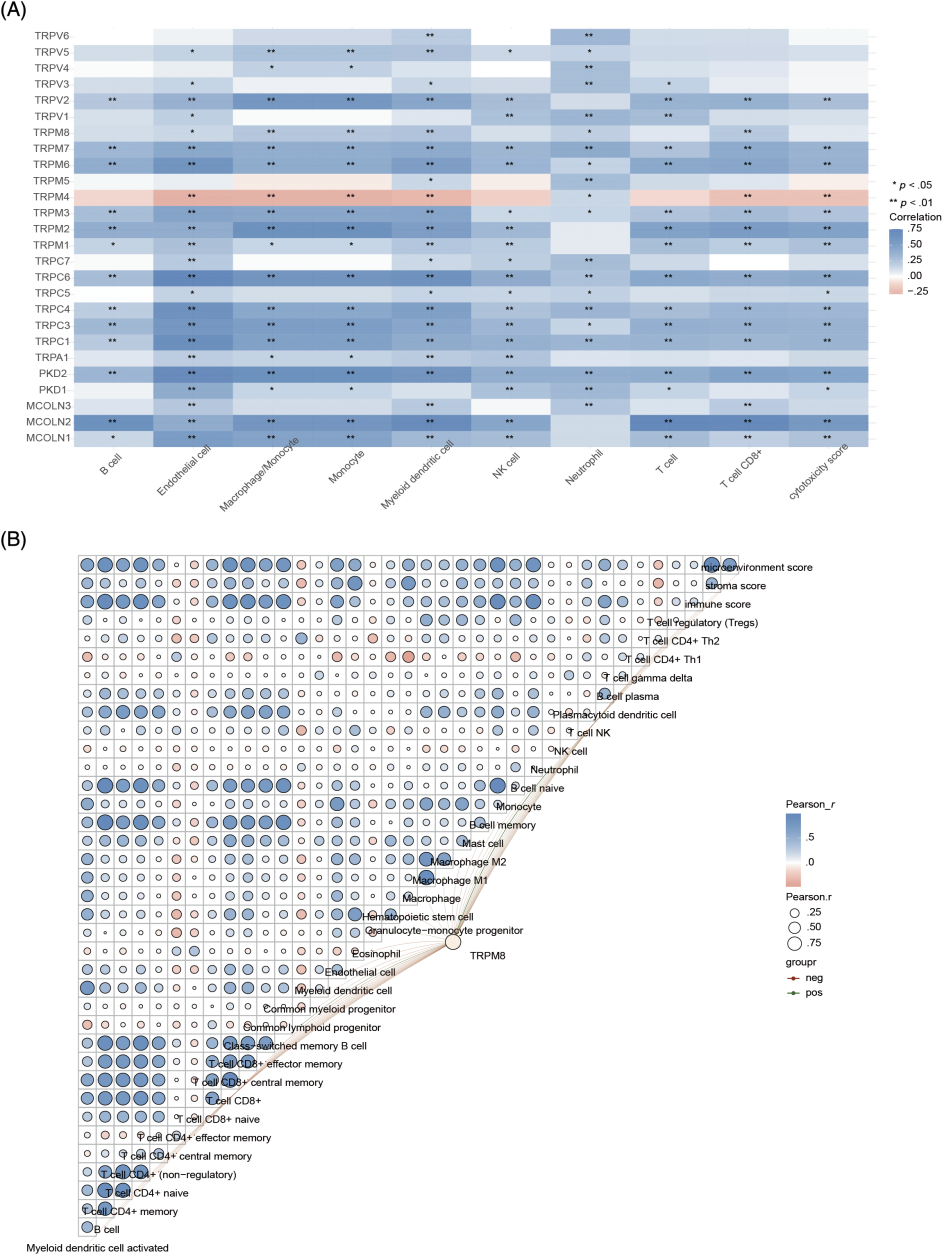
The correlations among multiple TRP genes. (A) A heat map of the correlation between multiple genes or models and immune score. The abscissa and ordinate represent genes, different colors represent different correlation coefficients (blue represents positive correlation whereas red represents negative correlation), the darker the color, the stronger the relation. Asterisks (*) stand for significance levels, ** for *p* < .01, * for *p* < .05. (B) The heat map in the schematic represents the correlation analysis of the immune score itself, red represents positive correlation, blue represents negative correlation, the more red or blue color means the greater correlation, also the larger circle means the stronger correlation; the red line in the schematic represents the negative correlation between the model score or gene expression and the immune score, green means the positive correlation. RNA‐sequencing expression profiles (level 3) and corresponding clinical data for XX individuals were downloaded from the TCGA dataset (https://portal.gdc.cancer.gov). The counts data were converted to Transcripts Per Million (TPM) and normalized using log2(TPM + 1), while ensuring the inclusion of samples with clinical information. Subsequently, XX samples were retained for further analysis. To validate the robustness of immune score assessment, we utilized immuneeconv, an R software package that integrates six state‐of‐the‐art algorithms: TIMER, xCell, MCP‐counter, CIBERSORT, EPIC, and quanTIseq. Each algorithm has been rigorously benchmarked, offering distinct advantages. Analysis and visualization were conducted using the ggClusterNet package within the R software environment. All aforementioned analytical approaches and R packages were implemented using R software version v4.1.3 (R Foundation for Statistical Computing, 2022). A significance level of *p* < .05 was considered statistically significant.

Initially, we conducted an investigation into the clinical correlation between TRP channels and immune subgroups of PAAD tumors utilizing the Timer database. Our findings revealed that a majority of TRP channels exhibited a positive association with immune infiltrated cells within PAAD tumors (Figure 7A). Specifically, TRPM8 demonstrated a strong connection with endothelial cells, monocyte‐macrophages, myeloid dendritic cells, neutrophils, and T cells CD8+. To elaborate further, TRPM8 displayed predominantly positive associations with T cell CD4+ memory, T cell CD4+ naive, T cell CD4+ (nonregulatory), T cells CD8+, T cells CD8+ central memory, T cells CD8+ effector memory, macrophage M1, and macrophage M2 via XCELL (Figure 7B). In this study, the infiltration of immune cells was evaluated utilizing algorithms such as MCPCOUNTER, CIBERSORT, and QUANTISEQ to bolster the reliability of the findings. A significant association was observed between TRPM8 and immune cells, specifically T cells and macrophages. This correlation was particularly notable in the case of CD8+ T cells and M2 macrophages (Figure S3). In previous research, it has been observed that macrophages play a role in aiding cancer cells in evading immune system attacks. Additionally, the expression of TRPM8 has been found to increase as PAAD progresses, suggesting a potential correlation.

### 3.5 Functional analysis of TRPM8 in PAAD

**FIGURE 8:**
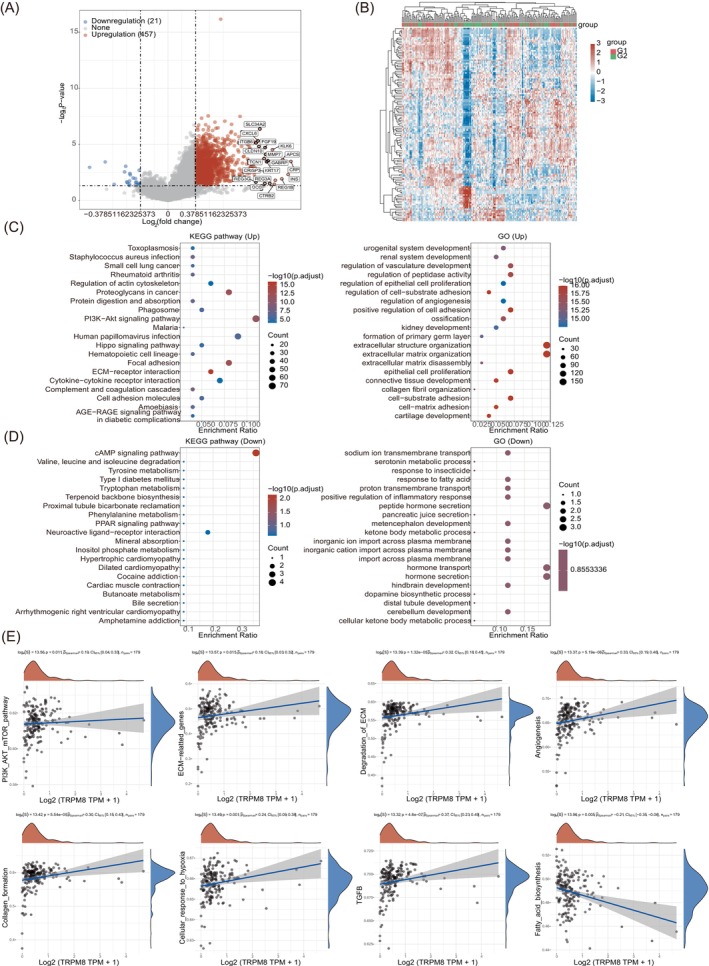
Functional analysis of TRPM8 in pancreatic adenocarcinoma (PAAD). (A) Differential gene expression in PAAD, which was regulated by high TRPM8 level. The top 20 genes were marked. Each dot represents one gene, red for upregulated genes, blue for downregulated genes, and gray for undifferentially expressed genes. (B) The top 50 differential genes were shown with the heat map in PAAD. The level of TRPM8 expression was employed as the criterion for classification, with G1 denoting the group with high TRPM8 expression and G2 representing the group with low TRPM8 expression. (C) KEGG pathway influenced by TRPM8 in PAAD (upregulated). The upregulated GO term by high TRPM8 in PAAD. The abscissa represents the enrichment ratio, and the ordinate shows the pathway names. The redder the color, the greater the significance. (D) KEGG analyzed downregulated functions in PAAD influenced by TRPM8. GO terms are used to analyze downregulated functions in PAAD influenced by TRPM8. (E) The correlations between individual gene and pathway score were analyzed with Spearman. The abscissa represents the distribution of the gene expression, and the ordinate represents the distribution of the pathway score. The density curve on the right represents the trend in distribution of pathway immune score, the upper density curve represents the trend in distribution of the gene expression. The value on the top represents the correlation *p* value, correlation coefficient and correlation calculation method. RNA‐sequencing expression profiles (level 3) and associated clinical data for PAAD subjects were acquired from the TCGA dataset (https://portal.gdc.cancer.gov). The ggstatsplot package in R software was utilized to visualize the relationships between gene expression and immune scores, while the pheatmap package in R was employed to illustrate multi‐gene correlations. Spearman's correlation analysis was conducted to depict the associations between nonnormally distributed quantitative variables. Statistical significance was determined by *p* values less than 0.05 (*p < .05). All analytical techniques and R packages were executed using R software.

To further investigate this relationship, patients with PAAD were categorized into groups based on their TRPM8 expression levels (high or low). Differential gene analysis, with a fold change greater than 2 and a *p*‐value less than .05, revealed distinct gene expression patterns influenced by the TRPM8 gene in PAAD, as depicted in the volcano plot (Figure 8A). Furthermore, RT‐PCR was performed to validate gene expression including TRPM8, SLC34A2, CXCL6, ITGB6, FGF19, and MMP7in PAAD tissues, which were consistent with the volcano plot analysis in bioinformatics (Figure S4 and Table S1). The heat map illustrated in Figure 8B showcases the top 50 genes that are upregulated and downregulated under the influence of the TRPM8 gene. A functional analysis of these significantly differential genes revealed that, among the upregulated genes, KEGG analysis indicated TRPM8's role in enhancing the cell proliferation‐related PI3K‐AKT signaling pathway in PAAD. Moreover, GO analysis suggested that the functions linked with genes upregulated by TRPM8 in PAAD predominantly involve the regulation of extracellular structure organization, extracellular matrix organization, cell proliferation, peptidase activity regulation, cell–matrix adhesion, and other related processes (Figure 8C). Our findings suggest a potential mechanism whereby elevated TRPM8 expression in PAAD could enhance cell proliferation, possibly through the upregulation of the PI3K‐AKT signaling pathway. Additionally, we undertook KEGG pathway and GO analyses to explore the functional consequences of genes downregulated by TRPM8 in PAAD (Figure 8D). The results indicated a significant correlation with the cAMP signaling pathway, sodium ion transmembrane transport, hormone secretion, and transport in relation to PAAD occurrence and progression. Furthermore, high TRPM8 expression was found to activate the PI3K‐AKT–mTOR pathway, ECM‐related genes, ECM degradation, angiogenesis, collagen formation, cellular response to hypoxia, and TGFβ. On the other hand, high TRPM8 expression appeared to downregulate fatty acid biosynthesis (Figure 8E). The analysis of downregulated genes in the GO enrichment pathway also suggested that highly expressed TRPM8 primarily impacts PAAD onset and progression by inhibiting various metabolic processes, particularly fatty acid biosynthesis.

**FIGURE 9:**
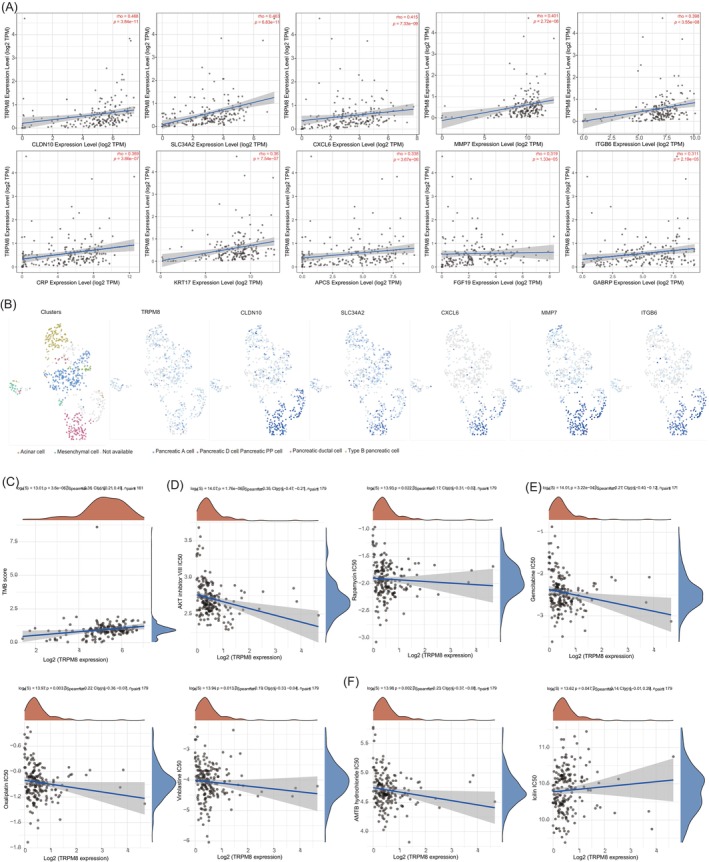
Correlation analysis of TRPM8 with other genes and drugs in pancreatic adenocarcinoma (PAAD). (A) Correlation analysis between TRPM8 and the top 10 genes (CLDN10, SLC34A2, CXCL6, MMP7, ITGB6, CRP, KRT17, APCS, FGF19, and GABRP). (B) The coexpression of TRPM8 and other genes was analyzed in single cells. (C) Correlation analysis between TRPM8 expression and tumor mutation burden (TMB) was performed using Spearman's method. The abscissa represents gene expression distribution, and the ordinate represents TMB score distribution. The density curve on the right represents the distribution trend of TMB score; the upper density curve represents the distribution trend of gene expression. The values on the top represents the correlation *p* value, correlation coefficient, and correlation calculation method. (D–F) Spearman correlation analysis of IC50 score and TRPM8 expression. The abscissa represents different groups of samples, and the ordinate represents the distribution of the IC50 score. The density curve on the right represents the trend in distribution of the IC50 score, the upper density curve represents the trend in distribution of the gene expression. The value on the top represents the correlation *p* value, correlation coefficient and correlation calculation method. (D) The correlation of TRPM8 and PI3K‐AKT–mTOR signaling pathway inhibitors including AKT inhibitor and Rapamycin in PAAD; (E) The effects of PAAD related drugs (Gemcitabine, Oxaliplatin, and Vinblastine) on TRPM8. (F) The correlation of TRPM8 and TRPM8 inhibitors including AMTB and Icilin in PAAD.

In Figures 8A and 9A, an analysis was conducted on the top 10 genes (CLDN10, SLC34A2, CXCL6, MMP7, ITGB6, CRP, KRT17, APCS, FGF19, and GABRP) regulated by TRPM8. Among these genes, CLDN10, SLC34A2, CXCL6, MMP7, and ITGB6 exhibited relatively high correlations with TRPM8 (*R* > .4). The results from single‐cell sequencing demonstrated that TRPM8 was predominantly colocalized with CLDN10, SLC34A2, CXCL6, MMP7, and ITGB6 in the duct cells of PAAD (GSE154778) (Figure 9B). Tumor mutation burden (TMB) has demonstrated potential as a predictive biomarker for various applications, including the correlation between different TMB levels and the response to immune checkpoint inhibitor treatment in diverse cancer patients. In this study, we have identified a significant association between a high level of TRPM8 and the TMB score, which is considered a predictive biomarker in PAAD (Figure 9C). Additionally, we have observed that TRPM8 upregulates the cell proliferation‐related pathway, specifically the PI3K‐AKT–mTOR signaling pathway (Figure 8C). Furthermore, the expression of TRPM8 was inhibited by AKT inhibitor VIII and mTOR inhibitor (Rapamycin) (Figure 9D). In this study, it was observed that PAAD‐related drugs such as Gemcitabine, Oxaliplatin, and Vinblastine exhibited a downregulation in the expression of TRPM8 (Figure 9E). Additionally, the effects of TRPM8 inhibitor (AMTB hydrochloride) and agonist (Icilin) were investigated and depicted in Figure 9F. AMTB hydrochloride was found to inhibit the expression of TRPM8, while Icilin was observed to promote its expression.

## 4 DISCUSSION

In this study, an initial analysis was conducted to examine the expression of the TRP family in patients with PAAD using bioinformatics methods. The results revealed an increased expression level of TRPM8. Subsequently, it was observed that higher expression levels of TRPM8 were associated with poor OS, PFS, DSS, and DFS. Additionally, the methylation status of TRPM8 was found to be significantly elevated, suggesting that methylation may contribute to the upregulation of TRPM8 expression, consequently, leading to reduced patient survival. Furthermore, analysis of immune cell infiltration showed that TRPM8 indicated a positive correlation with T cell subsets, including T cell CD4+ memory, T cell CD4+ naive, T cell CD4+ (nonregulatory), T cells CD8+, T cells CD8+ central memory, T cells CD8+ effector memory, and also associated with macrophage M1, and macrophage M2. TRPM8 facilitated the activation of cell proliferation‐associated pathways, particularly the PI3K‐AKT–mTOR signaling pathway, predominantly observed in ductal cells of PAAD. However, the upregulation of TRPM8 expression in PAAD patients resulted in enhanced cancer cell proliferation, evasion of immune surveillance, and expedited disease progression. Survival analysis further demonstrated that TRPM8 exerted a significant influence on the prognosis of PAAD patients.

PAAD, characterized by its high invasiveness, represents one of the most lethal malignancies. Since the survival rate of PAAD patients is low and the quality of life is severely reduced, the diagnosis and treatment of PAAD are very difficult in clinical practice. Therefore, we urgently need to find a biomarker that can specifically target PAAD. Currently, the number of drugs available for treating PAAD is very limited, many are in preclinical studies. For the treatment of pancreatic cancer, besides surgical techniques, chemotherapy, and palliative care, the research on targeted therapy, immunotherapy, and microbial therapy has gradually deepened.^28^ At present, two schemes are commonly used in the clinic: the combination of 5‐fluorouracil (5‐FU), irinotecan, leucovorin, and oxaliplatin, and the combination of gemcitabine and an albumin nanoparticle conjugate of paclitaxel (nab‐paclitaxel).^12^, ^29^, ^30^ In addition, studies have also shown that the combination of macrophage membrane coating (MPGNPs) and erlotinib synergistically inhibits the proliferation of pancreatic cancer cells by targeting Ras/Raf/MEK/ERK and PI3K/AKT/mTOR signaling pathways.^31^

The TRP channel family is a class of nonselective cation channels capable of allowing the permeation of Na^+^, Ca^2+^, Mg^2+^, and K^+^ through. These channels are widely expressed in diverse tissues and organs, and play a role in various pathophysiological processes. As sensory receptors, TRP channels can be activated by various natural plant preparations, leading to calcium influx and propagation of action potentials in primary sensory neurons. Consequently, they contribute to the transmission of temperature and pain signals in vivo. TRPV1, TRPA1, and TRPM8 are recognized as prominent receptors for capsaicin, mustard, and menthol, respectively, inducing sensations of burning, stinging, and cooling.^32^, ^33^ To inhibit TRP channels to block nociceptive transmission, two straightforward approaches can be employed: the inhibition of TRP channel activity through antagonists or the desensitization of TRP channels using agonists.^34^, ^35^, ^36^ In a randomized controlled experiment, the selective TRPV1 antagonist, ABT‐102, exhibited notable efficacy in significantly and reversibly elevating the thermal pain threshold and mitigating the suprathreshold pain perception.^37^ Furthermore, empirical research has demonstrated that the utilization of a high concentration (8%) capsaicin patch yields significant pain alleviation for individuals experiencing postherpes zoster pain and nerve injury pain associated with HIV infection.^38^ Additionally, scientific evidence has substantiated the pain‐relieving properties of menthol in countering chemical stimulation and inflammation through a TRPM8‐dependent mechanism.^39^, ^40^, ^41^ Notably, the absence of analgesic effects of menthol in TRPM8 knockout mice and the complete absence of analgesia induced by WS‐12 (a selective TRPM8 agonist) further support these findings.^42^, ^43^ The potential antimigraine properties of butterbur may be attributed to the selective activation of the TRPA1 channel by Isopetasin, the primary constituent of its extract, leading to subsequent notable neuronal desensitization.^44^ The TPR channel presents itself as a promising area of investigation for analgesic drug development, and further exploration of its involvement in tumor‐induced pain holds potential benefits for affected patients.

Conventional surgical procedures, chemotherapy, and radiotherapy, along with other established approaches for tumor treatment, are constrained by factors such as tumor staging, multidrug resistance, and reduced sensitivity. However, emerging immunotherapy, targeted therapy, and ion channel therapy offer promising prospects for patients with tumors. The TRP channel, functioning as a calcium ion permeable channel, has the potential to influence the initiation and progression of malignant tumors through the regulation of calcium homeostasis. Consequently, this prompts us to consider the feasibility of employing the TRP channel as an efficacious target for tumor treatment.^45^ TRPM8 was initially discovered in prostate tissue, and due to its upregulation in prostate cancer, it is regarded as a promising candidate for the diagnosis and treatment of prostate cancer. Subsequent research has demonstrated that the TRPM8 channel is overexpressed in various other tumor tissues, encompassing bladder cancer, human osteosarcoma, melanoma, squamous cell carcinoma, breast cancer, colorectal cancer, and pancreatic cancer.^46^ Chinigo et al. conducted a study that revealed the inhibitory effects of the TRPM8‐Rap1A interaction on cell migration and adhesion in prostate cancer and breast cancer.^47^ Differently, in cervical cancer, the overexpression of TRPM8 was found to have a greater impact on cell viability. Additionally, previous research suggests that TRPM8 may function as a testosterone receptor.^48^, ^49^ The results of the relevant experiments demonstrate that testosterone has the ability to bind and activate the TRPM8 protein implying that TRPM8 may play a role in the physiological and pathological mechanisms underlying testosterone dependence, potentially functioning as a testosterone receptor. Furthermore, the utilization of a TRPM8 antagonist has been shown to effectively impede the proliferation of androgen‐dependent prostate cancer cells,^50^ while concurrently diminishing the migratory and invasive properties of these tumor cells. The cytoplasmic C‐terminal of TRPM8 facilitates the interaction between TRPM8 and AMPK, thereby modulating the function of AMPK and regulating basal autophagy of cells through the AMPK‐ULK1 signaling pathway. TRPM8 potentially influences the proliferation and migration of breast cancer cells by modulating autophagy.^51^ Treatment with AMTB, a TRPM8 inhibitor, can diminish the proliferation and migration of breast cancer cells by targeting TRPM8‐related mechanisms. Additionally, AMTB may exert its effects on breast cancer cell lines with low TRPM8 expression by inhibiting voltage‐gated sodium channels.^52^ The research conducted by Wang et al. demonstrates that the inhibition of TRPM8 can impede the Akt‐GSK‐3β pathway and the phosphorylation of p44/p42 and FAK by disrupting intracellular calcium homeostasis.^53^ Consequently, this disruption hinders the progression of the cell cycle and suppresses the proliferation and growth of human osteosarcoma cell lines. Furthermore, the knockdown of TRPM8 also intensifies the apoptosis of osteosarcoma cells induced by epirubicin. The present study demonstrates a significant upregulation of TRPM8 expression in bladder cancer tissues. Subsequent knockdown of TRPM8 using specific siRNA transfection or inhibition of TRPM8 activity through BCTC (a TRPM8 antagonist) effectively suppressed the proliferation and migration of bladder cancer cells.^54^ Furthermore, these interventions were found to potentially modulate the ROS metabolism of bladder cancer cells via the MAPK and AKT/GSK3β signaling pathway, as well as the PPARγ‐SIRT1 feedback loop. Importantly, in vivo experiments provided evidence that knockdown of TRPM8 successfully impeded the growth of bladder cancer tissue. A research investigation on colorectal cancer has revealed that the overexpression of TRPM8 is indicative of an unfavorable prognosis for patients with colorectal cancer. Furthermore, the elimination or obstruction of TRPM8 may potentially diminish the growth of colorectal cancer by impeding the activation of the Wnt/β‐catenin signal transduction pathway.^41^

By studying the expression level and functional analysis of TRP channels in PAAD, we found and determined the abnormal expression of TRPM8 in PAAD and its pro‐proliferative role. TRPM8 expression levels were abnormally elevated in pancreatic cancer cells, and TRPM8 expression levels varied across substages of pancreatic cancer staging.^55^ Similarly, the analysis of TRP expression in patients with PAAD in our study suggests that TRPM8 expression levels are elevated. Overexpression of TRPM8 could upregulate the proliferation of pancreatic cancer cells by promoting cell cycle progression, and at the same time, the upregulated TRPM8 level could also enhance the invasive ability of pancreatic cancer cells.^56^ Similarly, Nelson et al. determined the expression level of TRPM8 in pancreatic cancer cells by real‐time PCR, and the results showed that TRPM8 expression was upregulated. A reduction in the proliferative progression ability of pancreatic cancer cells was shown after silencing TRPM8 with SiRNA.^57^ Functional analysis revealed that TRPM8 may promote PAAD proliferation through the PI3K‐AKT–mTOR signaling pathway, and further subsequent analysis showed that AKT inhibitor VIII and mTOR inhibitor (rapamycin) were able to downregulate TRPM8 expression. This finding is expected to provide insights into the signaling mechanism of pancreatic cancer cell proliferation and provide new hypotheses on the involvement of related ligands (stimuli or drugs that can affect TRPM8 activity) in influencing pancreatic cancer development. Related prognostic analyses showed a negative correlation between TRPM8 and the prognosis of PAAD patients, which is consistent with the potential proliferative mechanisms described above, suggesting the potential value of TRPM8 as a prognostic marker for PAAD. Additional studies have demonstrated that the LCK‐14‐3‐ζ‐TRPM8 axis influences the onset and progression of pancreatic cancer through the regulation of TRPM8 channel functionality, with LCK (lymphocyte‐specific protein tyrosine kinase) exerting a positive regulation channel function for TRPM8. LCK has been found to enhance the multimerization of TRPM8 through its promotion of the interaction between 14 and 3‐3ζ and TRPM8, thereby facilitating the regulation of TRP channel function.^58^ Silencing TRPM8 leads to a downregulation of the expression and activity of multidrug resistance‐related proteins such as P‐gp, MRP‐2, and LRP, as well as an impact on the levels of gemcitabine metabolism‐related proteins such as hENT1 and RRM1.^55^ In comparison with gemcitabine monotherapy, the coadministration of TRPM8 siRNA transfection and gemcitabine treatment demonstrates a substantial decrease in the cell viability of pancreatic cancer cells. This suggests a potential involvement of TRPM8 in the multidrug resistance exhibited by tumor cells.

## 5 CONCLUSION

By utilizing bioinformatics, our study successfully identified the aberrant expression of TRPM8 in PAAD and its pro‐proliferative effects through the PI3K‐AKT–mTOR axis, and identified TRPM8 as the most promising biomarker in the TRP family for the diagnosis and treatment of PAAD. Survival analysis suggested that the expression level of TRPM8 showed a negative correlation with the prognosis of PAAD patients, showing encouraging prognostic value. Given the cognitive level and comprehension constraints of the researchers, as well as the limitations of the funding support, our study has some limitations. First, our analysis of the results inevitably involves a certain degree of subjectivity, which may make the conclusions biased. The second is that our data and conclusions lack experimental support and clinical study verification, which is our future endeavor. According to our findings, the abnormal expression of TRPM8 is expected to provide value and significance for early diagnosis and prognostic analysis of PAAD patients to guide the adjustment of clinical strategies. In addition, the use of TRPM8 as a therapeutic target and the use of ligands to alter the activity of TRPM8 to guide the anticancer treatment of PAAD, in which PI3K‐AKT–mTOR may serve as a potential mechanism of TRPM8 anticancer therapy, provides a new direction for drug opening. We hope that our study will provide new insights into the diagnosis or treatment of pancreatic cancer to improve the efficacy and prognosis of pancreatic cancer patients.

## AUTHOR CONTRIBUTIONS

**Sen Qiao:** Conceptualization (lead); data curation (lead); formal analysis (lead); software (lead); writing – original draft (equal). **Fengming Wu:** Methodology (equal); supervision (equal); validation (equal); writing – original draft (equal). **Hongmei Wang:** Funding acquisition (supporting); project administration (lead); resources (lead); supervision (lead); writing – original draft (lead); writing – review and editing (lead).

## FUNDING INFORMATION

This work was financially supported by the Zhishan Scholars Programs of Southeast University (2242021R41070) and Fundamental Research Funds for the Central Universities (xzy012023127).

## CONFLICT OF INTEREST STATEMENT

The authors have stated explicitly that there are no conflicts of interest in connection with this article.

## ETHICS STATEMENT

All procedures were approved and performed in accordance with the ethic regulations committees of Southeast University (2022–66).

## Supporting information


**Data S1.** Supporting Information.
**Figure S1.** The function of all TRPs in PAAD. (A) The correlation between TRPM8, TRC6 and other family members. (B) The interactions between the TRP family and other genes using the GeneMANIA online website. (C, D) GO analysis and signaling pathway enrichment analysis of TRP family.
**Figure S2.** An analysis of TRPM8 expression and overall survival status in PAAD was conducted using the ICGC database. (A) The ICGC dataset comprises gene expression, survival time, and survival status. The top scatterplot illustrates the gene expression, which ranges from low to high, with different colors signifying various groups. The distribution of the scatter plot portrays the correlation of gene expression with survival time and survival status across different samples. The figure at the bottom represents the heat map of gene expression. (B) The Kaplan–Meier survival analysis of the gene signature derived from the ICGC dataset was employed, with a comparison conducted among diverse groups using the log‐rank test. HR (High exp) signifies the hazard ratio of the sample with low‐expression relative to the sample with high‐expression. A HR value greater than 1 suggests that the gene is a risk factor, whereas a HR value less than 1 indicates that the gene is a protective factor. HR (95% Cl) represents the median survival time (LT50) for various groups. (C) The ROC curve of the gene is depicted. Higher values of AUC are indicative of superior predictive power.
**Figure S3.** Correlation analysis of immune scoring. The heat map depicted in the diagram illustrates the correlation analysis of the immune score. A positive correlation is represented by red, while blue signifies a negative correlation. The intensity of the correlation is indicated by the depth of the color, with deeper red or blue signifying a stronger correlation. Additionally, the size of the circle also denotes the strength of the correlation, with larger circles representing stronger correlations. The red line in the diagram denotes a negative correlation between the model score or gene expression and the immune score, whereas green signifies a positive correlation.
**Figure S4.** RT‐PCR was performed to validate gene expression in PAAD tissues.
**Table 1.** Single‐cell RT‐multiplex PCR primers.

## Data Availability

Data sharing is not applicable to this article as no new data were created or analyzed in this study.
