# Supplementary material for: Genetic and immune identification and functional analysis of TRPM8 as a potential biomarker for pancreatic adenocarcinoma proliferation
Source: Cancer Rep (Hoboken). 2024 Jun 4;7(6):e2108. doi: 10.1002/cnr2.2108 (PMC11150080; doi:10.1002/cnr2.2108)
Supplement: Supplementary file 1 — Data S1. Supporting Information. Figure S1. The function of all TRPs in PAAD. (A) The correlation between TRPM8, TRC6 and other family members. (B) The interactions between the TRP family and other genes using the GeneMANIA online website. (C, D) GO analysis and signaling pathway enrichment analysis of TRP family. Figure S2. An analysis of TRPM8 expression and overall survival status in PAAD was conducted using the ICGC database. (A) The ICGC dataset comprises gene expression, survival time, and survival status. The top scatterplot illustrates the gene expression, which ranges from low to high, with different colors signifying various groups. The distribution of the scatter plot portrays the correlation of gene expression with survival time and survival status across different samples. The figure at the bottom represents the heat map of gene expression. (B) The Kaplan–Meier survival analysis of the gene signature derived from the ICGC dataset was employed, with a comparison conducted among diverse groups using the log‐rank test. HR (High exp) signifies the hazard ratio of the sample with low‐expression relative to the sample with high‐expression. A HR value greater than 1 suggests that the gene is a risk factor, whereas a HR value less than 1 indicates that the gene is a protective factor. HR (95% Cl) represents the median survival time (LT50) for various groups. (C) The ROC curve of the gene is depicted. Higher values of AUC are indicative of superior predictive power. Figure S3. Correlation analysis of immune scoring. The heat map depicted in the diagram illustrates the correlation analysis of the immune score. A positive correlation is represented by red, while blue signifies a negative correlation. The intensity of the correlation is indicated by the depth of the color, with deeper red or blue signifying a stronger correlation. Additionally, the size of the circle also denotes the strength of the correlation, with larger circles representing stronger correlation [file CNR2-7-e2108-s001.docx]

**Supplemental Materials**

**Figure S1.**


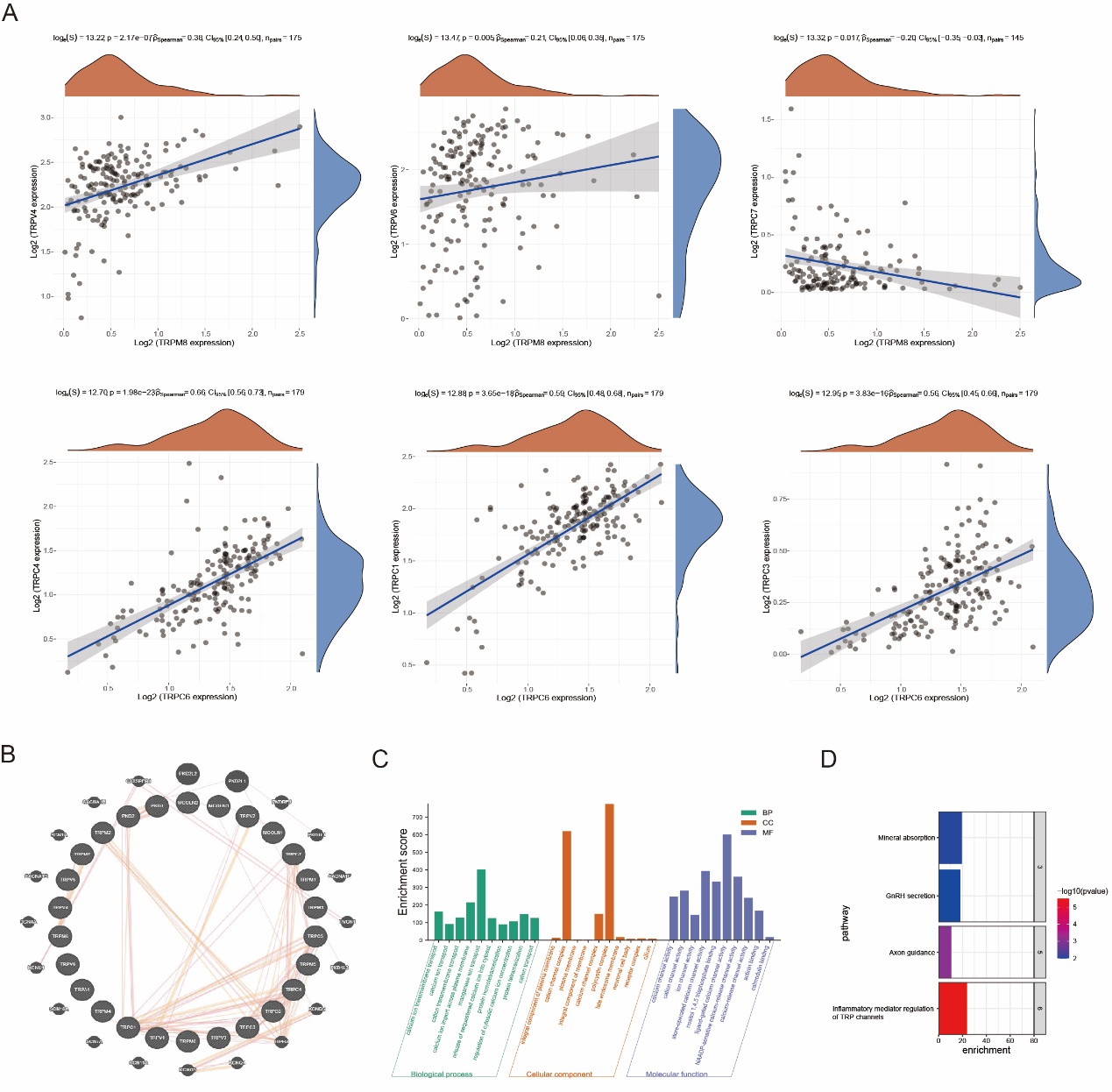


**Figure S1. The function of all TRPs in PAAD.**

(A) The correlation between TRPM8, TRC6 and other family members. (B) The interactions between the TRP family and other genes using the GeneMANIA online website. (C-D) GO analysis and signaling pathway enrichment analysis of TRP family.

**Figure S2**


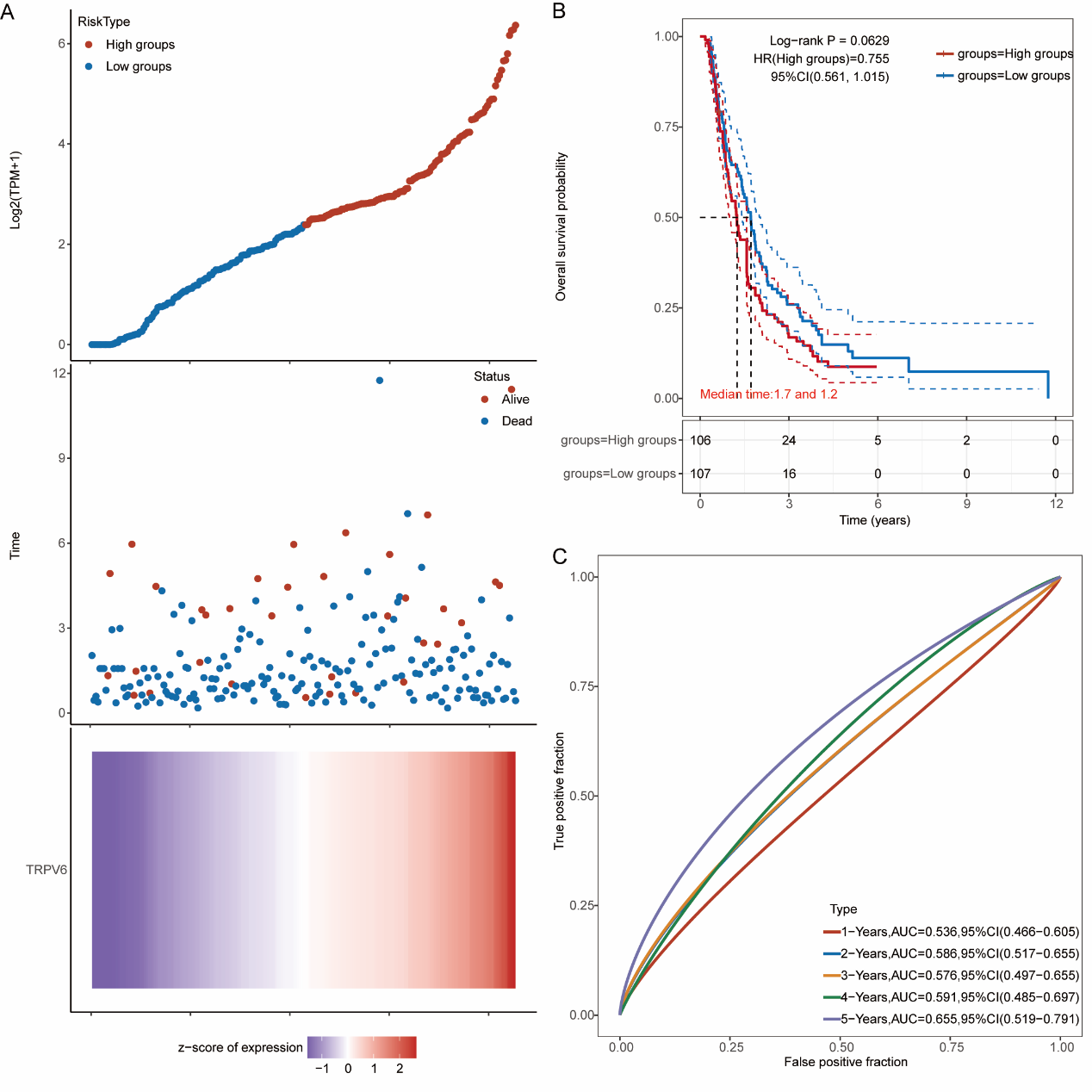


**Figure S2. An analysis of TRPM8 expression and overall survival status in PAAD was conducted using the ICGC database.** (A) The ICGC dataset comprises gene expression, survival time, and survival status. The top scatterplot illustrates the gene expression, which ranges from low to high, with different colors signifying various groups. The distribution of the scatter plot portrays the correlation of gene expression with survival time and survival status across different samples. The figure at the bottom represents the heatmap of gene expression. (B) The Kaplan-Meier survival analysis of the gene signature derived from the ICGC dataset was employed, with a comparison conducted among diverse groups using the log-rank test. HR (High exp) signifies the hazard ratio of the sample with low-expression relative to the sample with high-expression. A HR value greater than 1 suggests that the gene is a risk factor, whereas a HR value less than 1 indicates that the gene is a protective factor. HR (95%Cl) represents the median survival time (LT50) for various groups. (C) The ROC curve of the gene is depicted. Higher values of AUC are indicative of superior predictive power.

**Figure S3.**


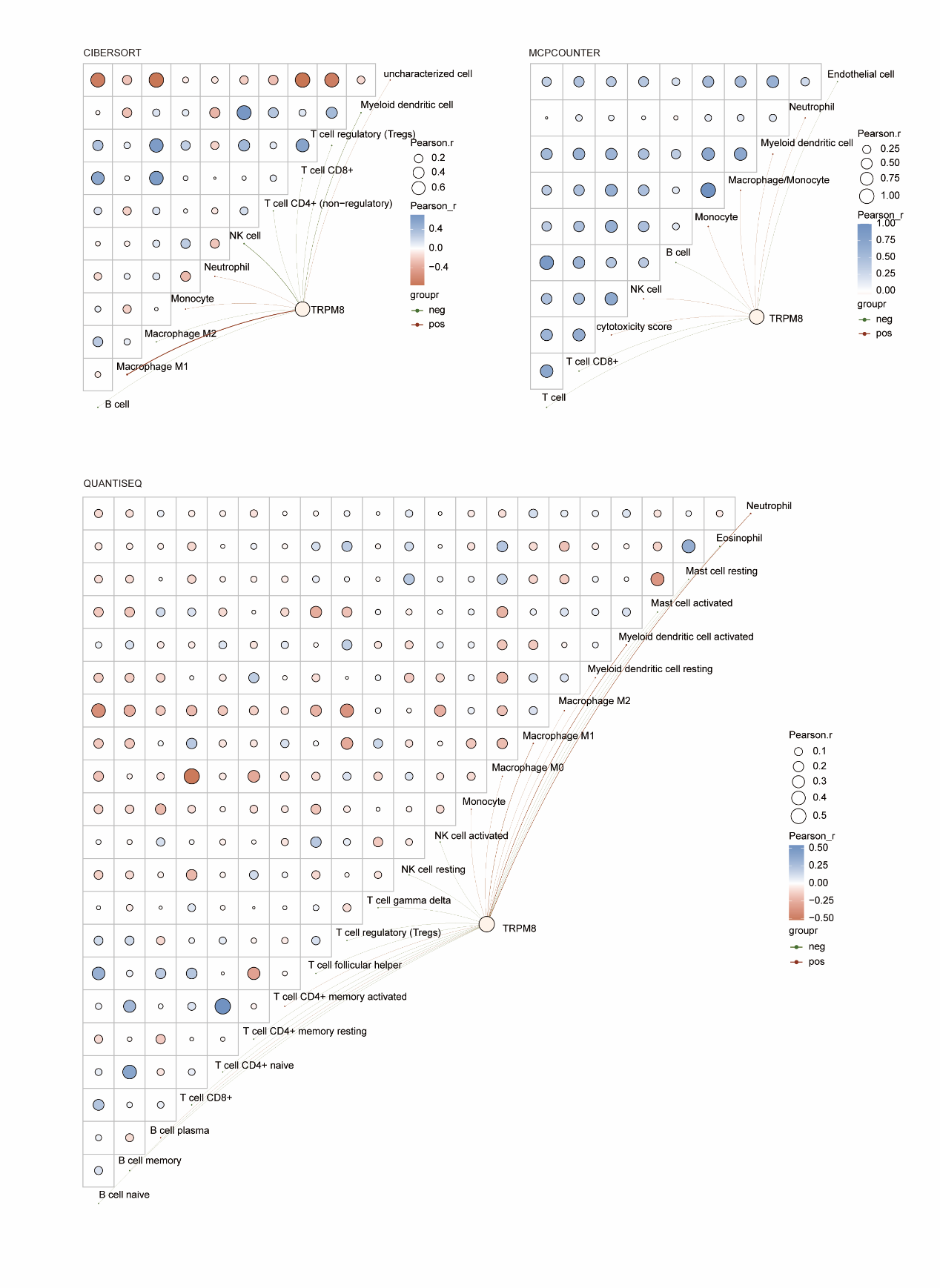


**Figure S3. Correlation analysis of immune scoring.** The heat map depicted in the diagram illustrates the correlation analysis of the immune score. A positive correlation is represented by red, while blue signifies a negative correlation. The intensity of the correlation is indicated by the depth of the color, with deeper red or blue signifying a stronger correlation. Additionally, the size of the circle also denotes the strength of the correlation, with larger circles representing stronger correlations. The red line in the diagram denotes a negative correlation between the model score or gene expression and the immune score, whereas green signifies a positive correlation.

**Figure S4.**


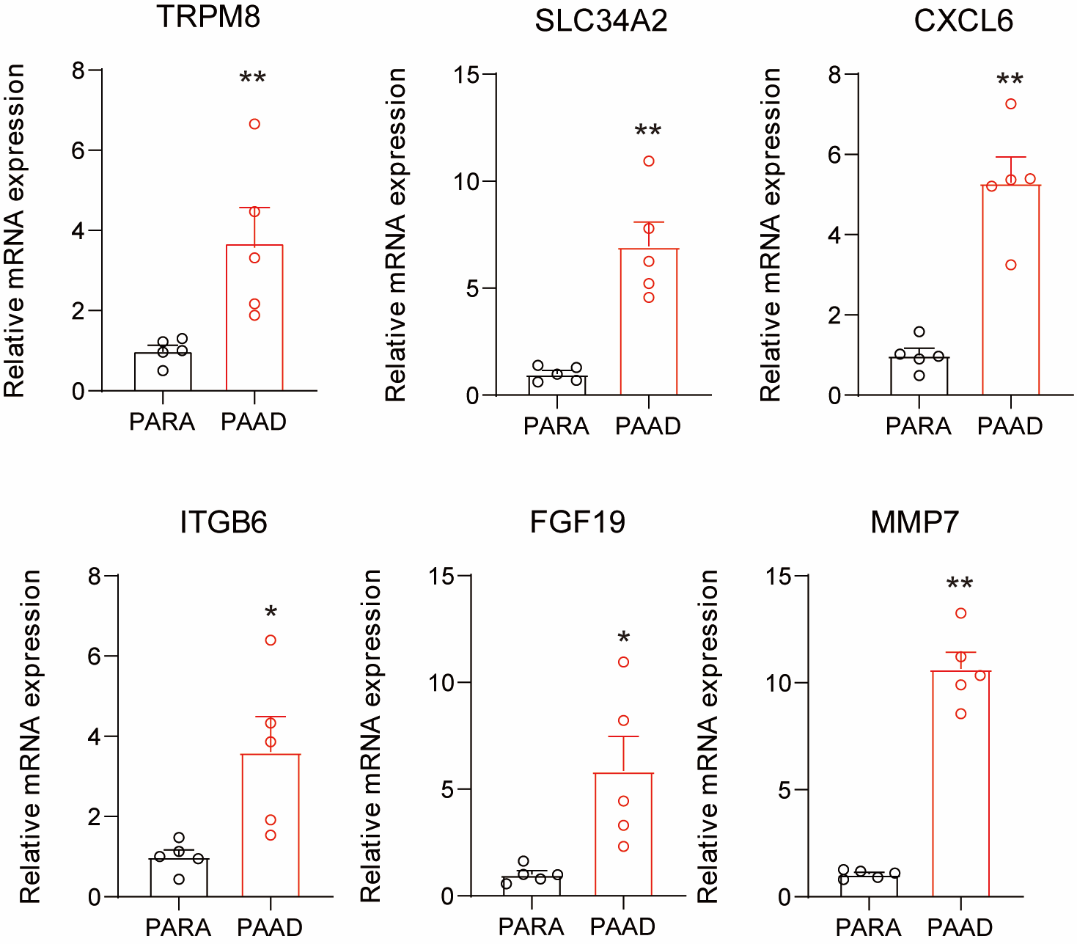


**Figure S4. RT-PCR was performed to validate gene expression in PAAD tissues.**

Table SA. Single-cell RT-multiplex PCR primers

| **Names** | **Forward Primer (5’ to 3’)** | **Reverse primer (5’ to 3’)** | **Amplified product (bps)** | **Accession** |
| --- | --- | --- | --- | --- |
| *TRPM8* | ATGACACTCTGGACAGCACC | CTCCGTGGCCTTGGAATCTT | 152 | NM_001397606.1 |
| *SLC34A2* | AACGTCACTGTTCCCTCGAC | GAGGACCAGCAGGGAGAGTA | 192 | NM_001177998.2 |
| *CXCL6* | TGCGTTGCACTTGTTTACGC | CCGTTCTTCAGGGAGGCTAC | 121 | NM_002993.4 |
| *ITGB6* | GGAGGTGCAGAAACCTGTGA | TCTGCCTACACTGAGAGGCT | 207 | NM_000888.5 |
| *FGF19* | CAGAGCGCGCACAGTTTG | GCGGATCTCCTCCTCGAAAG | 162 | NM_005117.3 |
| *MMP7* | AGTGGTCACCTACAGGATCGTA | GGCCCATCAAATGGGTAGGA | 195 | NM_002423.5 |
